# Elucidating the metabolic pathway and initial degradation gene for *p*-chloro-*m*-xylenol biodegradation in *Rhodococcus pyridinivorans* DMU114

**DOI:** 10.1128/aem.00984-25

**Published:** 2025-08-18

**Authors:** Liang Zhao, Jia Shi, Jingwei Wang, Hao Zhou, Dan Xu, Qiao Ma

**Affiliations:** 1 College of Environmental Science and Engineering, Institute of Environmental Systems Biology, Dalian Maritime University 12421, Dalian, China; 2 School of Chemical Engineering, Ocean and Life Science, Dalian University of Technology 506954 , Panjin, China; Danmarks Tekniske Universitet The Novo Nordisk Foundation Center for Biosustainability, Kgs. Lyngby, Denmark

**Keywords:** *p*-chloro-*m*-xylenol, biodegradation, *Rhodococcus*, flavin-dependent monooxygenase, metabolic pathway

## Abstract

The antimicrobial agent *p*-chloro-*m*-xylenol (PCMX), an emerging environmental pollutant, poses ecological risks; however, its biodegradation mechanisms remain unresolved. Here, we elucidate the metabolic pathway and functional genes involved in the initial catabolic step of PCMX in a newly isolated bacterium, *Rhodococcus pyridinivorans* DMU114. Pure-culture and synthetic consortium assays confirmed the pivotal role of *Rhodococcus* in PCMX degradation, despite its relatively low abundance in the PCMX-enriched consortium. Genomic analysis and heterologous expression identified a constitutively expressed flavin-dependent monooxygenase CxyAB as the key enzyme initiating PCMX degradation. High-resolution liquid chromatography-mass spectrometry and nuclear magnetic resonance analyses demonstrated that strain DMU114 degraded PCMX via a potential three-step pathway: *ortho*-hydroxylation to 4-chloro-3,5-dimethylcatechol, dechlorination to 2-hydroxy-3,5-dimethyl-[1,4]benzoquinone, and dual *meta*- and *ortho*-cleavage of the aromatic ring. Homologs of CxyA are phylogenetically widespread in environmentally relevant genera, including *Streptomyces*, *Pseudomonas*, *Klebsiella*, and *Rhodococcus*, indicating their potential role in natural PCMX attenuation. This work provides the first genetic dissection of PCMX mineralization, offering critical insights into its environmental fates and bioremediation strategies targeting antimicrobial contaminants.

**IMPORTANCE:**

The widespread use of the antimicrobial agent *p*-chloro-*m*-xylenol (PCMX) in consumer products has raised environmental concerns due to its aquatic toxicity. However, the microbial mechanisms driving its natural breakdown remain poorly understood. This study reveals how a newly isolated bacterium, *Rhodococcus pyridinivorans* DMU114, mineralizes PCMX, a process critical for mitigating its ecological risks. This study, for the first time, elucidates the PCMX’s complete degradation pathway and identifies the functional genes for its initial conversion step. The degradation gene identified is widespread in environmentally relevant bacteria, suggesting that natural ecosystems may already harbor the potential to neutralize PCMX contamination. These findings advance our ability to predict PCMX’s environmental fate and provide a foundation for engineering microbial solutions to combat antimicrobial pollution.

## INTRODUCTION

Heightened public health concerns have led to a growing incorporation of antimicrobial agents into household products to enhance health security (1). The COVID-19 pandemic further prompted a surge in disinfectant use (2). While triclosan (TCS) and triclocarban (TCC) were historically prevalent antimicrobials in personal care products (PCPs), they faced prohibitions in antiseptic wash products across developed nations, including the United States, since 2016 due to ecological and health risks (3). This regulatory shift facilitated *p*-chloro-*m*-xylenol (PCMX) adoption as a safer alternative. Marketed for its broad-spectrum efficacy and presumed lower toxicity, PCMX has dominated the PCP market through sustained consumption growth (1). For example, studies have indicated that PCMX is present in 16.9% of antiseptic detergents in the United States, 20.7% of household cleaners in the United Kingdom, and 33.9% of hand sanitizers in China (4). Nevertheless, its widespread environmental presence and inadequate removal during wastewater treatment processes have caused concerning contamination levels across multiple ecosystems (5–10). Emerging studies document PCMX concentrations ranging from μg/L to mg/L in wastewater facilities, freshwater systems, and coastal environments, underscoring its prolonged ecological consequences (11–13).

Initially considered safe, emerging evidence now reveals PCMX’s multifaceted ecological risks (14). Acute and chronic aquatic toxicity has been demonstrated across model species, inducing embryo malformation, mortality, developmental morphological abnormalities, and neurotoxicity in zebrafish (15), rainbow trout (16), and aquatic invertebrates (17). PCMX also adversely affects the life span and reproductive capacity and induces mitochondrial dysfunction in pseudocoelomate organisms, including *Brachionus koreanus* (18) and *Caenorhabditis elegans* (3). Upon environmental partitioning into soil and sludge matrices, PCMX readily interacts with microbes, where compelling evidence shows that it enhances antibiotic resistance gene (ARG) horizontal transfer efficiency (19). Concurrently, it also negatively affects bioreactor functionality and shifts microbial community structures (20). These adverse impacts highlight the urgent need for a thorough and comprehensive risk characterization of PCMX across environmental compartments (21).

Elucidating PCMX’s environmental fates and transformation pathways is critical for comprehensive risk assessment, yet its degradation processes in natural systems remain poorly characterized. Biodegradation is the key mechanism for PCMX transformation (22, 23). Pioneering work demonstrated the co-metabolic transformation of PCMX by *Aspergillus niger* in the presence of glucose, later expanded to *Cunninghamella elegans* and *Trametes versicolor* through putative cytochrome P450- and laccase-mediated mechanisms (24). Recent advances revealed *Rhodococcus* spp. as promising bacterial degraders with cross-laboratory validation. Several *Rhodococcus* strains, including PCMX2, DMU2021, GG1, and JH-7, have been successively isolated from sludge samples with superior degradation efficiency (22–26). *Rhodococcus* has an evolutionary advantage due to its large genomic repositories containing redundant catabolic pathways, which facilitate the mineralization of structurally diverse recalcitrant aromatics (27–30). This functional prowess positions *Rhodococcus* as a powerful genus for targeted bioremediation strategies.

Critical knowledge gaps persist in current PCMX biodegradation research. First, the complete biodegradation pathway of PCMX is unresolved, particularly regarding the contentious initial catalytic step. To date, only three studies have characterized the microbial transformation intermediates of PCMX. *Cunninghamella elegans* IM 1785/21GP and *Rhodococcus* sp. GG1 demonstrate chlorine-hydroxylation generating 2,6-dimethylhydroquinone, whereas *Rhodococcus* sp. JH-7 catalyzes *ortho*-hydroxylation to yield 4-chloro-3,5-dimethylcatechol (23–25). In addition, the downstream metabolic pathways are scarcely mentioned. Second, the functional genes responsible for PCMX degradation remain uncharacterized. Although the P450 enzyme has been proposed to be involved in PCMX hydroxylation based on qPCR and enzyme inhibition assay (23), the specific gene functions have yet to be validated. These uncertainties hinder our understanding of the PCMX environmental fates and metabolic mechanisms.

To address these gaps, we enriched a highly efficient PCMX-mineralizing consortium and isolated the novel strain *Rhodococcus pyridinivorans* DMU114. The catabolism processes and functional genes involved in PCMX degradation were systematically explored through the integrated amplicon sequencing, genomic sequencing, qRT-PCR profiling, heterologous gene expression, high-resolution liquid chromatography-mass spectrometry (HR-LCMS), and nuclear magnetic resonance (NMR) analyses. This study aims to (i) decipher consortium dynamics and *Rhodococcus*’ ecological partitioning in PCMX degradation, (ii) resolve the strain DMU114’s PCMX mineralization pathway, (iii) uncover the key genes involved in the first-step conversion of PCMX, and (iv) reveal the distribution of the functional genes.

## MATERIALS AND METHODS

### Chemicals and media

Mineral salt medium (MSM) contains (NH_4_)_2_SO_4_ 134 mg, KH_2_PO_4_ 141 mg, K_2_HPO_4_ 287 mg, MnSO_4_·H_2_O 2.68 mg, MgSO_4_·7H_2_O 21.4 mg, FeCl_3_·6H_2_O 0.134 mg, and CaCl_2_ 3.8 mg per liter of water. LB medium contains yeast extract 5.0 g, peptone 10.0 g, and NaCl 10.0 g per liter of water. Solid media were obtained by adding 2% (*w/v*) agar. All media were sterilized by autoclaving at 121°C for 20 min. PCMX was purchased from Sangon Biotech (Shanghai, China). A stock solution of PCMX was prepared by dissolving the compound in dimethyl sulfoxide.

### PCMX-degrading consortium enrichment

Soil samples were collected from the campus and transported to the laboratory for microbial acclimation. The acclimation procedure consisted of two sequential stages. In stage I, PCMX (50 mg L^−1^) was introduced as the sole carbon source into mineral salt medium (MSM), with weekly medium replenishment over a period of 4 months under controlled conditions (30°C, 150 rpm, pH 7.0 ± 0.2), yielding a PCMX-adapted microbial community (designated consortium A). In stage II, consortium A was sub-cultured through five successive transfers (5% [vol/vol] inoculum) into fresh MSM supplemented with 50 mg L^−1^ PCMX at 7-day intervals to selectively enrich PCMX-degrading microorganisms. The final consortium (consortium B) demonstrating superior PCMX degradation efficiency was obtained through this iterative enrichment strategy (Fig. S1).

### Characterization of microbial consortium

The original, consortium A, and consortium B samples were collected, centrifuged, and stored at −80°C. DNA extraction and high-throughput sequencing were conducted by Guangdong Magigene Biotechnology Co., Ltd. (Guangzhou, China). The V4 region of the bacterial 16S rRNA gene was amplified utilizing primers 515F (5′-GTGCCGCCGCGGTAA-3′) and 806R (5′-GGACTACHVGGGTWTCTAAT-3′), and sequencing was performed on the Illumina platform. The raw sequencing data were processed using the Galaxy platform (https://dmap.denglab.org.cn/) (6).

### Isolation of PCMX-degrading bacteria

The spread plate method was used to isolate bacteria from consortium B. The 16S rRNA genes of the isolated strains were amplified using universal primers (27F: 5′-AGAGTTTGATCCTGGCTCAG-3′; 1492R: 5′-GGTTACCTTGTTGTACGACTT-3′), and the sequences were compared in the EzBioCloud database. A phylogenetic tree was constructed using the neighbor-joining method in MEGA 11.0 software to evaluate the evolutionary relationship between strain DMU114 and related strains (31).

### PCMX biodegradation assay

PCMX degradation by strain DMU114 was carried out in the MSM with an inoculation size of 5% (vol/vol). Samples were taken at intervals to measure the residual PCMX by ultra-performance liquid chromatography (UPLC, Waters ACQUITY, USA) (22). Samples were treated with an equal volume of methanol and filtered through a 0.22 µm filter prior to analysis. The detection conditions were set as follows: mobile phase 35:65 (vol/vol) water/methanol, flow rate 0.8 mL min^−1^, column temperature 40°C, and wavelength 230 nm. The effects of various factors on PCMX degradation were investigated, including the initial PCMX concentration (10, 20, 40, 60, and 80 mg L^−1^), temperature (25, 30, 35, and 40°C), and pH (5.0, 6.0, 7.0, 8.0, and 9.0). To examine potential synergistic effects among the bacteria, the bacterial combination and artificial consortium assays were performed. For the combination assay, each of the isolated strains was cultured separately in LB medium, centrifuged, washed, and resuspended in phosphate-buffered solution (PBS, pH 7.0) to an optical density at 600 nm (OD_600_) of 1.0. These suspensions were then individually inoculated (1% [vol/vol]) into the MSM in combination with strain DMU114, and the PCMX degradation was monitored. For the artificial consortium assay, strains 2#, 3#, 4#, 5#, and 7# (the main populations in the PCMX-degrading consortium) were inoculated simultaneously and equivalently into MSM containing 50 mg L^−1^ PCMX as the sole carbon source. This consortium underwent five successive weekly transfers (5% [vol/vol]), and the bacterial community dynamics were determined.

### Genome sequencing

The genomic DNA of DMU114 was extracted and purified, and sequencing was processed using the PacBio Sequel IIe platform. Genome assembly was performed using the mecat2 tool after filtering and quality control of the sequencing data. The assembled sequences were annotated and analyzed using the Rapid Annotations using Subsystems Technology (RAST), NCBI Prokaryotic Genome Annotation Pipeline, Cluster of Orthologous Groups of Proteins (COG), Kyoto Encyclopedia of Genes and Genomes (KEGG), and Gene Ontology (GO) databases.

### Quantitative real-time RT-PCR (qRT-PCR) assay

The transcription levels of the P450 enzyme and catechol dioxygenase genes in strain DMU114 cultured in LB medium, with and without PCMX, were determined by qRT-PCR (32). Total RNA was isolated using the Bacteria RNA Extraction Kit (Vazyme, China). Reverse transcription-PCR was performed using HiScript II 1st Strand cDNA Synthesis Kit (Vazyme, China), and the resulting cDNAs were quantified with gene-specific primers using ChamQ SYBR qPCR Master Mix (Vazyme, China) on a real-time PCR system (LightCycler 480II, Roche, Switzerland). The *gyrB* gene was used as the reference gene. Primers were provided in Table S1.

### Heterologous expression of functional genes

The PCR-amplified products of the target genes were ligated into the linear pET-28a(+) PCR product vector using the ClonExpress II One Step Cloning Kit (Vazyme, China) through homologous recombination. Then, the recombinant plasmids were transformed into *E. coli* DH5 competent cells. Positive colonies were selected on LB agar plates with 50 mg L^−1^ kanamycin and verified by PCR and sequencing. The verified recombinant plasmids were transformed into *E. coli* BL21(DE3) for expression. As for the resting cell experiment, the recombinant *E. coli* BL21(DE3) strain was cultured in LB medium with kanamycin until reaching an OD_600_ of 0.4. Protein expression was induced with 0.2 mM isopropyl-β-dthiogalactopyranoside at 30°C. The bacterial cells were collected, washed, and resuspended in PBS (OD_600_ 2.0) to prepare the resting cell system. PCMX was added to start the reaction, and residual concentration was determined by UPLC. Primers for gene expression were provided in Table S2.

### Metabolite identification

Possible PCMX metabolites were analyzed by pooling samples collected at different time intervals and analyzed by HR-LCMS (Orbitrap Exploris 480, Thermo Fisher Scientific, Germany) (33). The chromatographic separation was achieved using a binary mobile phase system (water and acetonitrile) at a flow rate of 0.2 mL min^−1^ with column temperature 40°C. The gradient elution program was set as follows: 5% acetonitrile (vol/vol) (0–13 min), 5–100% acetonitrile (13–16 min), and 5% acetonitrile (16–18 min). The metabolite produced by the gene-engineering strain was further analyzed by NMR on a Bruker Avance NEO-400 spectrometer. Preparative HPLC separations were performed using a Novasep instrument and Waters auto-P and 2695 series instruments with a preparative C18CE column. The mobile phase consisted of 0.1% ammonium hydroxide in water (vol/vol) and 0.1% ammonium hydroxide in methanol (vol/vol). The elution gradient was as follows: 5% methanol (0–5 min), 5–95% methanol (5–50 min), and 95% methanol (50–60 min). The flow rate was 80 mL min^−1^ at room temperature.

### Molecular docking

Structural prediction of CxyA was performed through homology modeling against the crystallographically resolved (*S*)-3-chloro-β-tyrosine monooxygenase SgcC (PDB ID: 4OO2, 64.7% identity). The tertiary structure of CxyA was constructed using the Protenix server (https://protenix-server.com/), incorporating four flavin adenine dinucleotide (FAD) cofactors and four PCMX ligands into the homotetrameric assembly. Molecular docking simulations were visualized through PyMOL. Interactions between PCMX and amino acid residues were analyzed via the PLIP server (https://plip-tool.biotec.tu-dresden.de/plip-web/plip/index).

### Gene distribution analysis

A complete set of bacterial genomes (*N* = 36,754, to date March 2025) was retrieved from the National Center for Biotechnology Information (NCBI) RefSeq database to construct a microbial proteomic database (*N* = 333,218,737 protein sequences). Subsequently, this database was queried against the reference CxyA sequence (XVG13778) using DIAMOND (v2.1.11). Homologous sequences (amino acid identity >40%, query coverage >70%, and subject coverage >70%) were retrieved for the distribution analysis.

## RESULTS AND DISCUSSION

### Enrichment of the PCMX-degrading consortium

**Fig 1:**
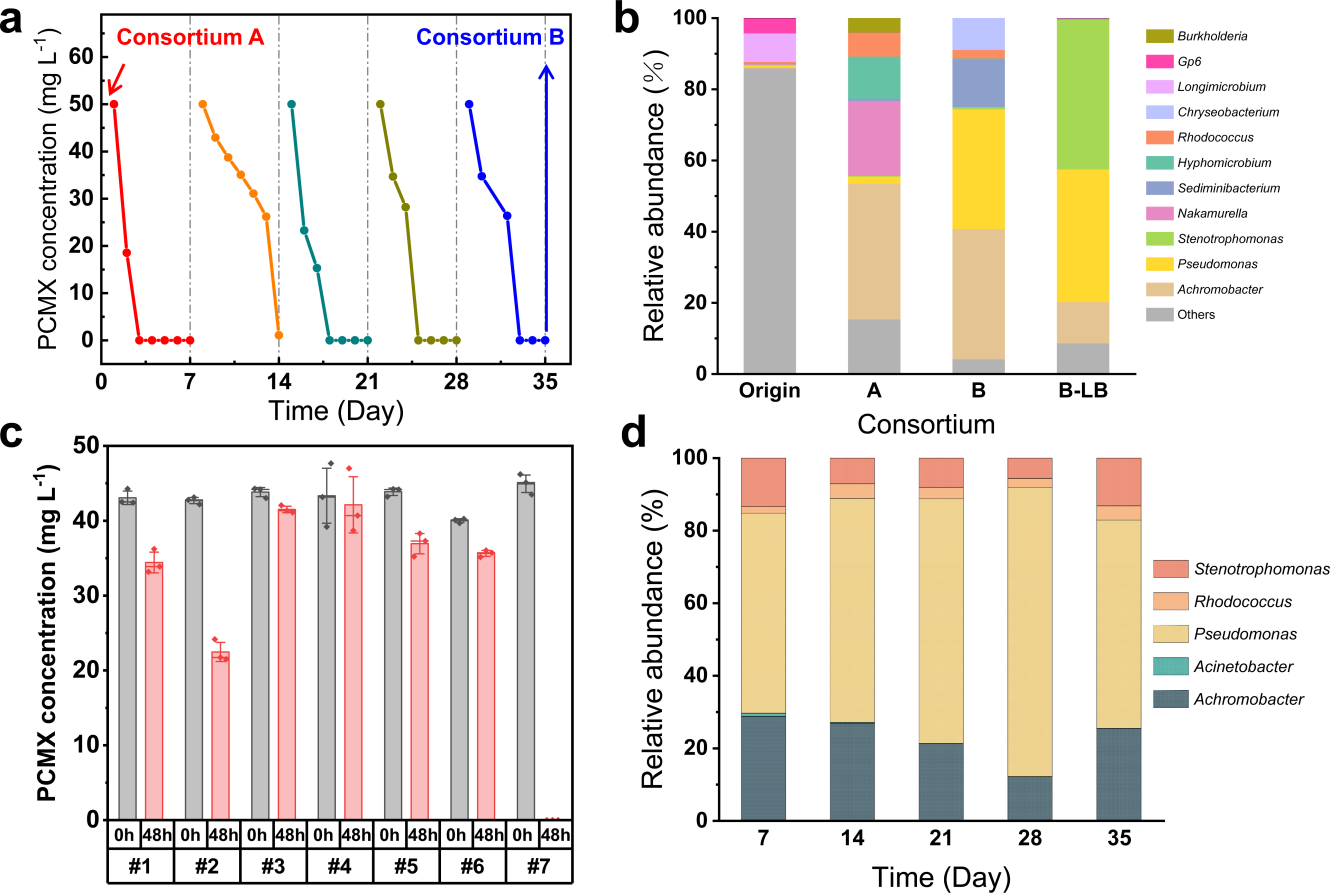
Functional and structural characterization of the PCMX-degrading microbial consortia. (**a**) Serial transfer cultivation (5% [vol/vol] inoculum) process from consortium A (initial inoculum) to consortium B (final consortium at day 35, Fig. S1). (**b**) Dynamics of the consortium microbial structure in the enrichment process at the genus level. B-LB meant that the consortium B was subcultured in LB medium for 24 hours. (**c**) PCMX degradation performance of the pure strains isolated from consortium B. The assay was conducted using LB medium. (**d**) Structural dynamics of a synthetic consortium containing five defined strains during weekly serial transfers in MSM. Equivalent numbers of pure strains *Acinetobacter* (strain #2), *Stenotrophomonas* (strain #3), *Pseudomonas* (strain #4), *Achromobacter* (strain #5), and *Rhodococcus* (strain #7) were mixed and cultured with a weekly transfer (5% [vol/vol] inoculum).

The soil sample was acclimated for four months to establish a stable and highly efficient sludge consortium (consortium A, Fig. S1) capable of PCMX degradation. Subsequent enrichment was performed through successive weekly transfers in MSM over five weeks, yielding consortium B. The microbial consortium consistently achieved complete degradation of 50 mg L^−1^ PCMX within seven days, maintaining stable degradation performance across multiple generations (Fig. 1a). Comparative analysis of microbial community structures revealed significant shifts. Taxonomic profiling identified 29, 14, and three phyla in the original sludge, consortium A, and consortium B, respectively. Consortium B exhibited substantial enrichment of Pseudomonadota (74.4%), Bacteroidota (23.2%), and Actinomycetota (2.4%) at the phylum level (Fig. S2). Genus-level analysis demonstrated a compositional shift from *Achromobacter* (38.1%), *Nakamurella* (21.1%), *Hyphomicrobium* (11.8%), *Rhodococcus* (6.6%), and *Burkholderia* (4.1%) in consortium A to *Achromobacter* (36.6%), *Pseudomonas* (33.6%), *Seminibacterium* (13.4%), *Chlorobacterium* (8.9%), and *Rhodococcus* (2.4%) in consortium B (Fig. 1b), suggesting the potential of these strains for PCMX degradation. Inoculation of consortium B (1% [vol/vol]) into nutrient-rich LB medium induced significant structural divergence from the MSM-adapted community, with *Stenotrophomonas* (42.1%), *Pseudomonas* (37.3%), and *Achromobacter* (11.6%) dominating the population—indicative of the superior nutrient utilization capacity of these strains.

Similar functional convergence occurred in PCMX-degrading consortia enriched from different activated sludges using PCMX as the sole carbon source, with *Achromobacter*, *Pseudomonas*, and *Rhodococcus* consistently emerging as the dominant genera (22, 25). However, reactor-acclimated sludge microbial communities fed with PCMX and glucose exhibited divergent taxonomic profiles dominated by *Azomonas* and *Tolumonas* (34), demonstrating that enrichment protocols critically determined consortium structures. Notably, the recurrent dominance of *Achromobacter*, *Pseudomonas*, and *Rhodococcus* underscored their pivotal roles in PCMX catabolism (22, 25).

### Isolation and characterization of the PCMX-degrading bacteria

Seven pure bacterial strains were isolated from consortium B (*Microbacterium* [#1], *Acinetobacter* [#2], *Stenotrophomonas* [#3], *Pseudomonas* [#4], *Achromobacter* [#5], *Bacillus* [#6], and *Rhocococcus* [#7], Table S3). Strain #2 demonstrated a 48.7% removal of 40 mg L^−1^ PCMX within 48 hours, while strain #7 achieved complete elimination (Fig. 1c). The remaining five strains exhibited negligible degradation capacity. This functional disparity necessitated a systematic investigation of interspecies interactions. Co-culture experiments combining strain #7 with other isolates demonstrated that *Pseudomonas* (strain #4) exerted distinct antagonistic effects on PCMX catabolism, while other strains showed no significant impacts (Fig. S3). These results confirmed that strain #7 exhibited superior catabolic activity in the degradation of PCMX. Furthermore, an artificial consortium was established through the equivalent inoculation of *Acinetobacter*, *Stenotrophomonas*, *Pseudomonas*, *Achromobacter*, and *Rhodococcus* in MSM containing PCMX as the sole carbon source. The coculture underwent five successive transfers at seven-day intervals, and the stabilized consortium exhibited structural convergence with consortium B, dominated by non-degrading populations of *Pseudomonas* (57.4%) and *Achromobacter* (25.4%), while the functionally critical *Rhodococcus* persisted at a lower abundance (3.9%) (Fig. 1d).

Our findings reveal an intriguing observation: while monoculture experiments showed no evidence of cooperative enhancement in PCMX degradation by non-*Rhodococcus* strains (Fig. S3), their persistent dominance in the synthetic consortium (Fig. 1d) suggested that they might play important ecological roles beyond direct degradation. This disparity could indicate that these high-abundance populations (e.g., *Pseudomonas* and *Achromobacter*) might contribute as ecological facilitators, potentially by mitigating intermediate toxicity, providing public goods, or engaging in cross-feeding to support community stability and function (35). Future integration of metatranscriptomic profiling with isotope tracing is required to elucidate the interactions between *Rhodococcus* and other non-degraders under more realistic conditions (36).

### Strain DMU114 is efficient in PCMX degradation

Strain #7, designated DMU114, exhibited an opaque and orange-yellow coloration with the smooth, convex, and moist colonial morphology. It presented a short rod-like morphology with a length of 1 µm (Fig. S4a). Phylogenetic analysis positioned strain DMU114 within *Rhodococcus pyridinivorans* (Fig. S4b). Recent studies have identified *Rhodococcus* spp. as key microbes for PCMX biodegradation, highlighting their ecological dominance and widespread distribution in PCMX-degrading microbial communities (22–26). Strain DMU114 exhibited effective degradation of PCMX at concentrations below 40 mg L^−1^, while further increases in PCMX concentration significantly impeded both microbial growth and degradation efficiency (Fig. S5). The degradation performance of PCMX by strain DMU114 was investigated under different conditions that followed the first-order kinetic model (Fig. S6). Theoretical half-lives *t*_1/2_ of PCMX (20 m L^−1^) were 18.1, 14.2, 13.5, and 20.7 hours at pH values of 6.0, 7.0, 8.0, and 9.0; and 17.4, 14.2, 9.8, and 48.5 hours at temperatures of 25, 30, 35, and 40°C, respectively (Table S4). These results illustrated that the optimal conditions were pH 6.0–8.0 and 25–35°C. The strain demonstrated biodegradation activity toward multiple PCMX derivatives, including 4-chlorophenol, *m*-cresol, 3,5-dimethylphenol, 2,6-dimethylbenzene-1,4-diol, and 2,4-dichloro-3,5-dimethylphenol (Fig. S7), indicating potential applicability in remediating complex phenolic contaminants.

### Identification of skatole degradation metabolites

Four possible intermediates were identified during the PCMX degradation with the compound as sole carbon source, and they were proposed as 3-hydroxy-2,4-dimethyl-hexa-2,4-dienedioic acid (P1, RT 4.44 min, C_8_H_10_O_5_, [M-H] 185.04544), 2,4-dihydroxy-3,5-dimethyl-6-oxo-hexa-2,4-dienoic acid (P2, RT 4.86 min, C_8_H_10_O_5_, [M-H] 185.04543), 2,4-dimethyl-3-oxo-hexanedioic acid (P3, RT 7.57 min, C_8_H_12_O_5_, [M-H] 187.09749), and 4-hydroxy-3-methyl-2-oxo-hex-4-enoic acid (P4, RT 6.25 min, C_7_H_10_O_4_, [M-H] 157.05063) (Fig. S8). These metabolites corresponded to the *ortho*- and *meta*-ring cleavage products of the catechols, consistent with established enzymatic pathways mediated by catechol 1,2-dioxygenase (C12O) and catechol 2,3-dioxygenase (C23O) in phenolic compound degradation (37–43).

To elucidate pre-cleavage metabolites, 2,2'-bipyridyl was employed to inhibit aromatic ring fission (44–46). This strategy enabled the identification of 2-hydroxy-3,5-dimethyl-[1,4]benzoquinone (P5; RT 7.91 min; C_8_H_8_O_3_; [M-H] 151.03995) (Fig. S9), demonstrating dechlorination preceded ring cleavage—a metabolic pattern shared with strain GG1 (23). The proposed degradation pathway is initiated with PCMX hydroxylation at C2 and C4 positions, leading to the formation of 2-hydroxy-3,5-dimethyl-[1,4]benzoquinone. It was further degraded via both C12O and C23O pathways, ultimately channeling metabolites into the tricarboxylic acid cycle (Fig. S10). Nevertheless, the initial transformation product was not detected, which is likely attributable to its rapid subsequent conversion.

### Genome features of strain DMU114

The complete genome of strain DMU114 comprised a single circular chromosome (5,241,731 bp, G+C content 67.9%), with 5,070 predicted protein-coding genes spanning 4,760,529 bp (90.8% genome coverage) (Fig. S11). Genomic features included eight sRNAs, 12 rRNAs, and 57 tRNAs. Comparative genomics revealed 97.9% average nucleotide identity (ANI) with *R. pyridinivorans* JCM 10940, demonstrating taxonomic classification within this species. Functional annotation through multiple databases revealed 4,933 (NR), 3,288 (Swiss-Prot), 3,921 (COG), 2,489 (GO), and 4,828 (KEGG) annotated genes (Fig. S12a). KEGG pathway analysis identified dominant metabolic functions, including carbon metabolism (133 genes), amino acid biosynthesis (117), ABC transporters (110), benzoate degradation (60), and aromatic compound degradation (45) (Fig. S12b). RAST subsystem annotation identified 85 genes associated with aromatic compound metabolism. Notably, 75 putative oxygenase genes were annotated, potentially relating to PCMX catabolism (Table S5).

### Identification of the potential gene clusters for PCMX degradation

**Fig 2:**
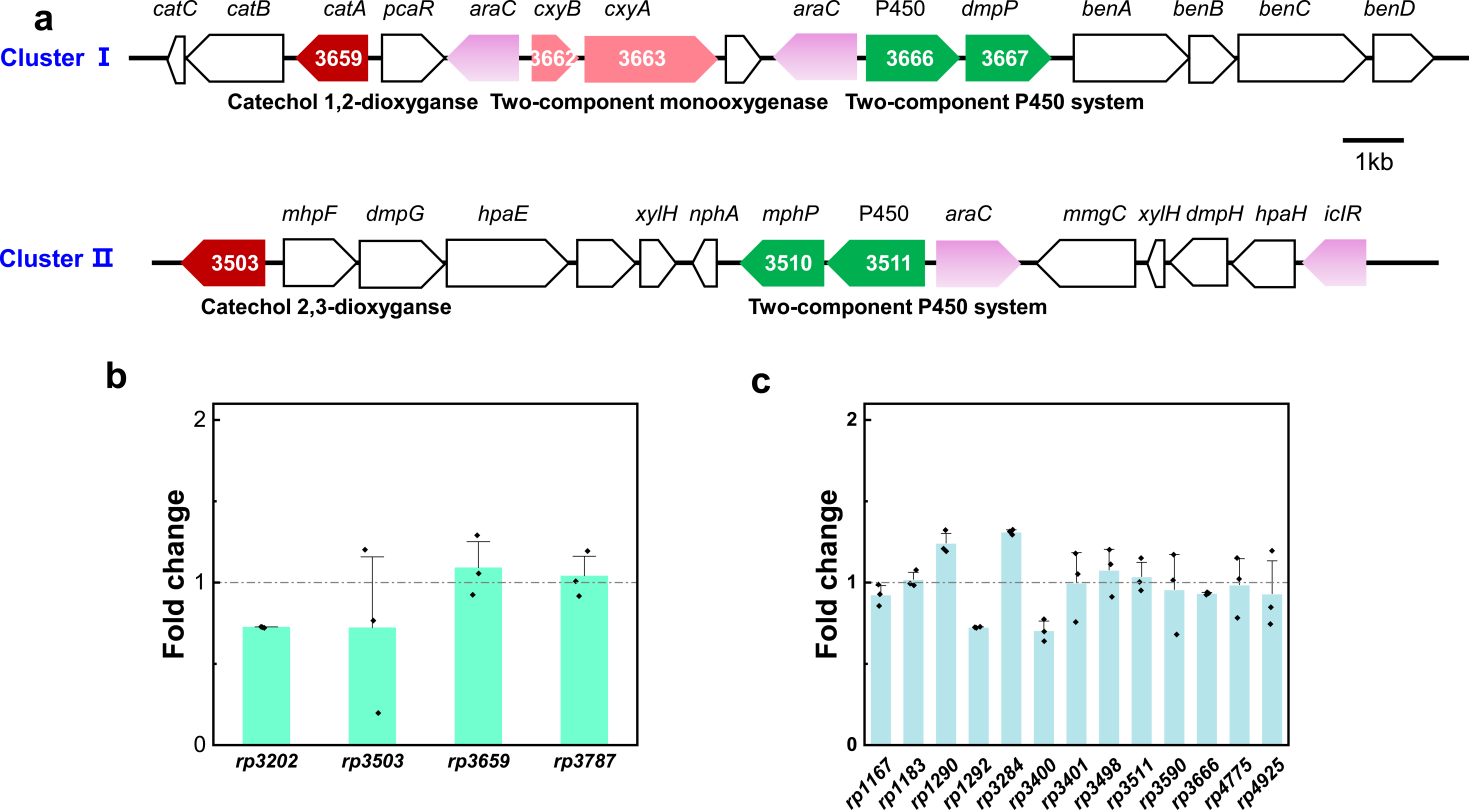
Potential genes for PCMX degradation in strain DMU114. (**a**) Proposed two functional gene clusters. Both clusters contain the P450 enzyme genes and catechol dioxygenase genes. (**b**) qRT-PCR results of the catechol dioxygenase genes. (**c**) qRT-PCR results of the P450 enzyme genes.

Previous studies reported that cytochrome P450 might play a crucial role in PCMX biodegradation (23). Genome analysis of strain DMU114 identified 13 putative cytochrome P450 genes and four catechol dioxygenases, i.e., three C12O genes (*rp3202*, *rp3659*, and *rp3787*) and one C23O gene (*rp3503*), which might participate in the downstream metabolism processes (Table S5 and S6) (37, 42). Aromatic-degrading gene clusters are often clustered (37, 47, 48). Coincidentally, the C12O gene *rp3659* and C23O gene *rp3503* co-localized with P450 oxygenase genes within two distinct catabolic clusters (Fig. 2a) (Table S7). Cluster I contained the *catABC* operon governing catechol *ortho*-cleavage, alongside three oxidoreductases: a cytochrome P450 (*rp3666*), benzoate 1,2-dioxygenase (*rp3668*), and putative 4-hydroxyphenylacetate 3-monooxygenase (*rp3663*). Cluster II featured P450-associated *meta*-cleavage components, including C23O (*rp3503*) and a complete C23O metabolic pathway comprising 5-carboxymethyl-2-hydroxymuconate semialdehyde dehydrogenase (*rp3506*), 4-oxalocrotonate tautomerase (*rp3514*), 4-oxalocrotonate decarboxylase (*rp3515*), 2-oxo-hepta-3-ene-1,7-dioic acid hydratase (*rp3516*), 4-hydroxy-2-oxovalerate aldolase (*rp3505*), and acetaldehyde dehydrogenase (*rp3504*). These enzymatic complements facilitated the complete mineralization of *meta*-cleavage products into the tricarboxylic acid cycle. The genomic co-occurrence of dual cleavage systems, supported by intermediate profiling (Fig. S10), confirmed strain DMU114’s capacity for both C12O- and C23O-mediated PCMX degradation pathways.

### The functional genes for PCMX degradation should be constitutively expressed

To verify the expression pattern of PCMX-degrading genes, resting cell experiments were performed (49). Strain DMU114 was cultured in the presence and absence of PCMX, harvested, resuspended in PBS (OD_600_ 2.0), and subsequently exposed to 40 mg L^−1^ PCMX to initiate the reaction. Strikingly, both induced and non-induced cells demonstrated comparable PCMX degradation efficiencies (Fig. S13), indicating the constitutive expression of catabolic genes. Complementary qRT-PCR analysis of all the catechol dioxygenase and cytochrome P450 genes revealed minimal transcriptional variation upon PCMX exposure compared to the control group (Fig. 2b and c), further confirming the constitutive expression patterns. This persistent enzymatic readiness likely confers ecological competitiveness in fluctuating contaminant niches.

### Genes *cxyAB* rather than CYP450 initiate PCMX degradation

**Fig 3:**
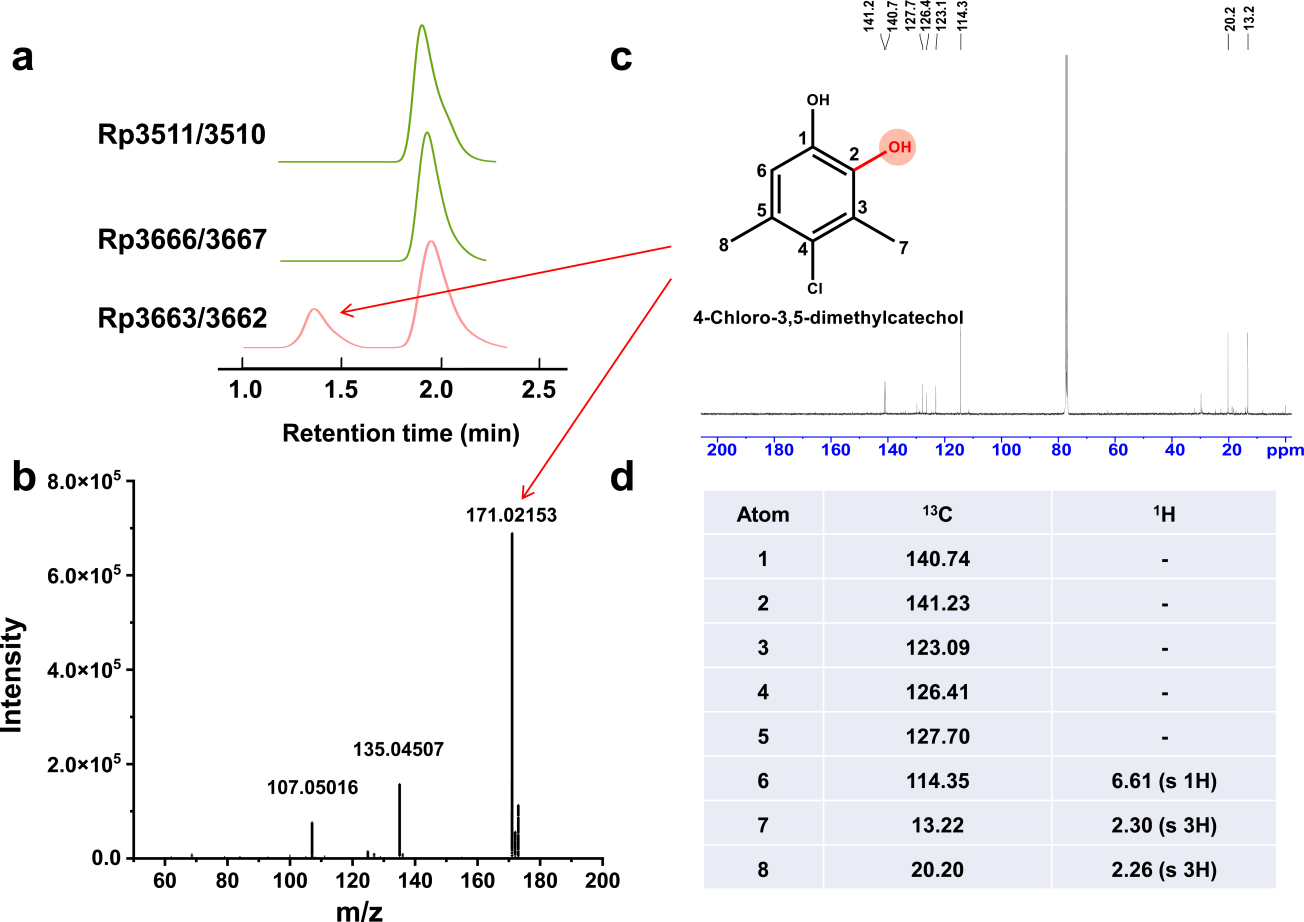
Characterization of the functional enzymes for PCMX initial degradation. (**a**) Catalytic potential of the expressed *rp3511/3510*, *rp3666/3667*, and *rp3663/3662* genes. (**b**) Mass spectrum of the catalytic product by recombinant strain expressing gene *rp3663/3662*. (**c**)^ 13^C NMR of the product. (**d**) Summary of the ^13^C and ^1^H information of the product.

Genomic mining and functional predictions revealed three putative enzymatic systems potentially initiating PCMX transformation, i.e., cytochrome P450 genes *rp3666/3667* (cluster I) and *rp3511/3510* (cluster II), as well as a 4-hydroxyphenylacetate 3-monooxygenase gene *rp3663/3662* (cluster I). Heterologous expression of these two-component enzymatic systems in *E. coli* BL21(DE3) was conducted. The recombinant cells were cultured, induced, and collected for the resting cell experiments, which demonstrated that *rp3663/3662* (here designated *cxyAB*) exclusively generated PCMX-derived metabolites, while P450-containing systems showed no catalytic activity (Fig. 3a). Meanwhile, we also tested the PCMX degradation performance of strain DMU114 in the presence of two P450 enzyme inhibitors, i.e., 1-aminobenzotriazole and piperonylbutoxide (Fig. S14) (50, 51). It was shown that 1-aminobenzotriazole did not affect the PCMX degradation performance. Slight inhibition of PCMX degradation was observed in the piperonylbutoxide group, which should be attributed to the suppression of strain DMU114 growth. The dual-line evidence excluded P450 enzyme-mediated hydroxylation as the initial PCMX transformation step. Their specific roles in the PCMX degradation process warranted future investigation.

### 4-Chloro-3,5-dimethylcatechol is the PCMX first-step catalytic product

Structural elucidation of the CxyAB-catalyzed product was achieved through HR-LCMS, revealing a molecular ion at *m/z* 171.02153 ([M-H]) with the characteristic chlorine isotopic signature. The molecular formula was C_8_H_9_ClO_2_, corresponding to the hydroxylation product of PCMX, which is consistent with 4-chloro-3,5-dimethylcatechol (Fig. 3b) (25). To further verify the structure, we performed a large-scale resting cell experiment (4 L) to obtain the reaction mixture, which was extracted and purified by preparative HPLC separations. The isolated compound (P6) was obtained as a white crystalline solid. Its chemical structural formula was deduced from its ^1^H and ^13^C NMR data (Fig. 3c and d). The 1D NMR data and HSQC spectra (Fig. S15) indicated characteristic resonances for one phenyl ring (δH 6.61 [1H, s], δC 140.74, 141.23, 123.09, 126.41, 127.70, 114.35) and two methyl groups (δH 2.30 [3H, s], δH 2.26 [3H, s], δC 13.22, 20.20). These spectral signatures validated the *ortho*-hydroxylation of PCMX to 4-chloro-3,5-dimethylcatechol.

### Proposed PCMX degradation pathway in strain DMU114

**Fig 4:**
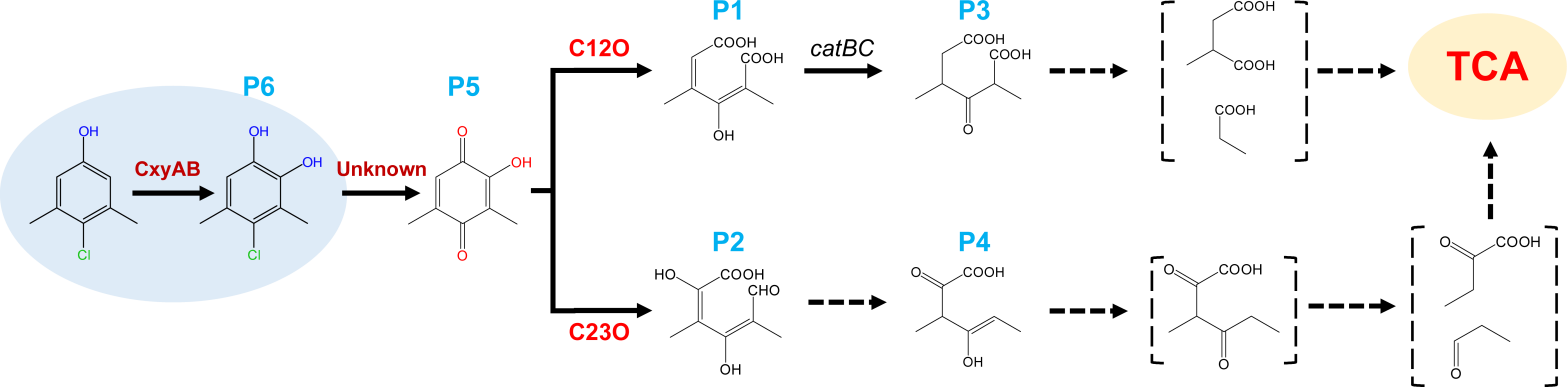
Proposed PCMX biodegradation pathway in strain DMU114.

By integrating metabolic profiling data and gene expression results, we refined the PCMX degradation pathway in strain DMU114 (Fig. 4). The transformation proceeded through sequential enzymatic steps (1). *ortho*-Hydroxylation of PCMX to 4-chloro-3,5-dimethylcatechol (2), dechlorination yielding 2-hydroxy-3,5-dimethyl-[1,4]benzoquinone, and (3) dual ring cleavage pathways mediated by C12O and C23O. This mechanistic clarification demonstrated that the initial step involved *ortho*-hydroxylation catalyzed by a flavin monooxygenase rather than dechlorination (23, 25). This catalytic step aligned with the findings observed in the strain *Rhodococcus* sp. JH-7, but differed from that of strain *Rhodococcus* sp. GG1, highlighting a strain-specific metabolic behavior. P450 enzymes represent a superfamily of oxidoreductases with versatile catalytic functions, including dehalogenation (52). Specifically, it has been suggested that the P450 enzyme catalyzed the hydroxylated dechlorination of PCMX in *Rhodococcus* sp. GG1 (23). Consequently, it is hypothesized that the dechlorination process (the second step) of PCMX degradation may be mediated by a P450 enzyme within the associated gene cluster. This hypothesis requires further investigation.

### CxyA belongs to the group D flavin-dependent monooxygenases

**Fig 5:**
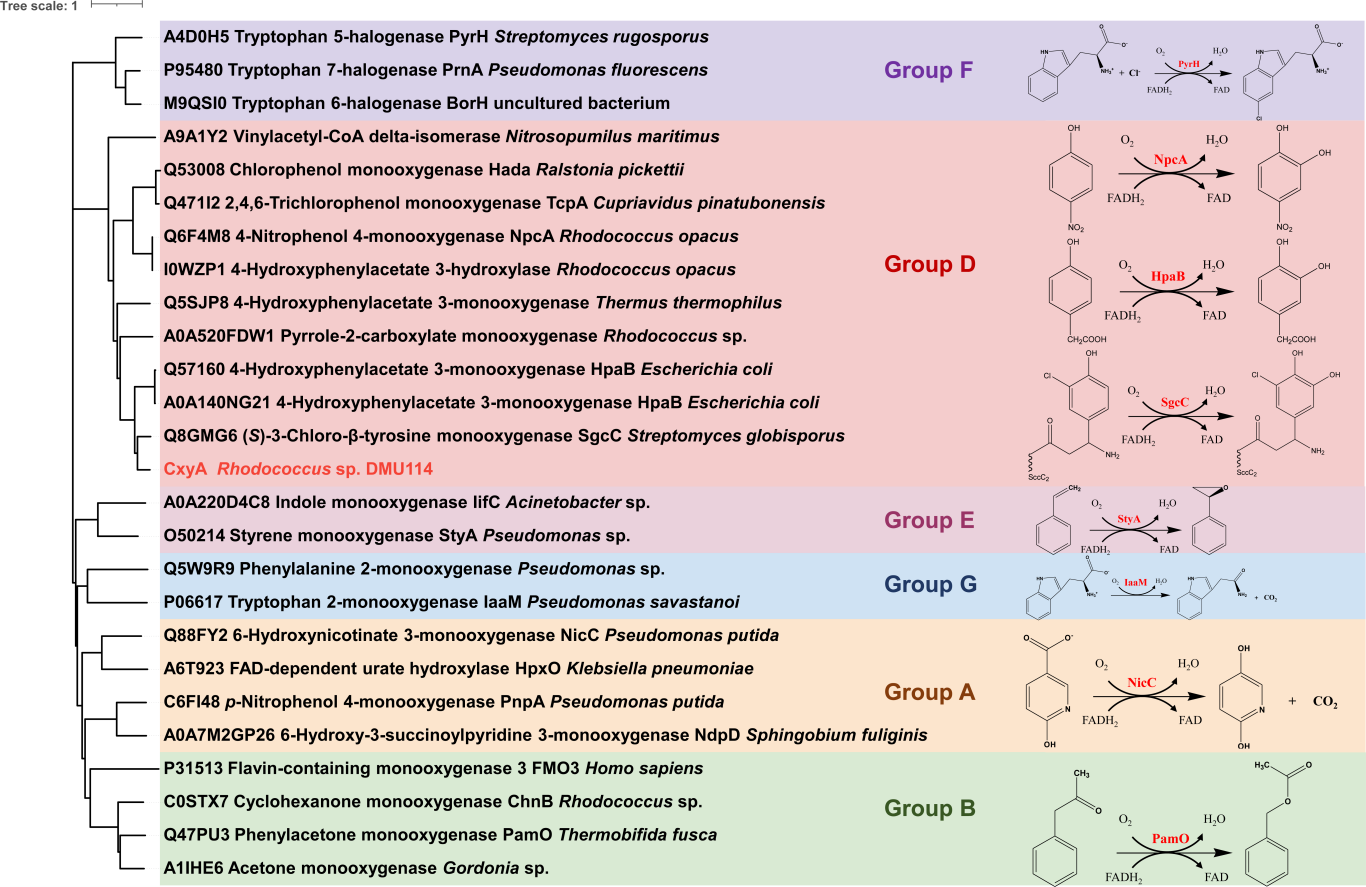
Phylogenetic tree of CxyA and related flavin-dependent monooxygenases.

To characterize CxyA’s functional attributes, we conducted phylogenetic analysis and multiple sequence alignment with biochemically validated homologs (53). The results classified CxyA as a group D flavin-dependent monooxygenase (54). It was closely related to the well-characterized (*S*)-3-chloro-β-tyrosine-S-SgcC2 hydroxylase SgcC from *Streptomyces globisporus* (64.7%), 4-hydroxyphenylacetate 3-monooxygenase HpaB from *Escherichia coli* (55.2%), and 4-nitrophenol 4-monooxygenase NpcA from *Rhodococcus opacus* (55.6%) (Fig. 5; Table S8) (55–58). While these enzymes catalyze phenolic substrate *ortho*-hydroxylation, their activity toward PCMX remains unconfirmed. Conserved substrate-binding residues (Arg135, Tyr139, and His177), critical for catalytic coordination, were identified through structural alignment (Fig. S16). The three-dimensional structure of CxyA-PCMX complex generated using the Proteinix server exhibited high-quality scores (pTM = 0.95, ipTM = 0.94), indicating the structural plausibility of the homotetrameric assembly (Fig. S17). Two distinct loop regions (Trp175-Asp195 and Ile229-Lys237) within CxyA were identified as critical for FAD binding (green motif, Trp175-Asp195) and PCMX interaction (orange motif, Ile229-Lys237), respectively. Specifically, PCMX binds to the active pocket formed by Tyr139, His177, Ile179, Tyr231, Leu312, Phe321, and Arg399. Hydrophobic interactions with residues Ile179, Ile180, Tyr231, Leu312, Phe317, and Phe321 further stabilize PCMX within the binding pocket. Sequence comparison with CxyB revealed its low similarities with the corresponding reductases SgcE6 (36.9%) from *S. globisporus*, HpaC (26.4%) from *E. coli*, and NpcB (32.5%) from *R. opacus*. Bioinformatics analyses revealed that CxyB should be a member of the NphA2-like flavin reductase subfamily. Further studies are required to fully elucidate the enzymatic properties of CxyA and CxyB.

### CxyA is widespread in nature

**Fig 6:**
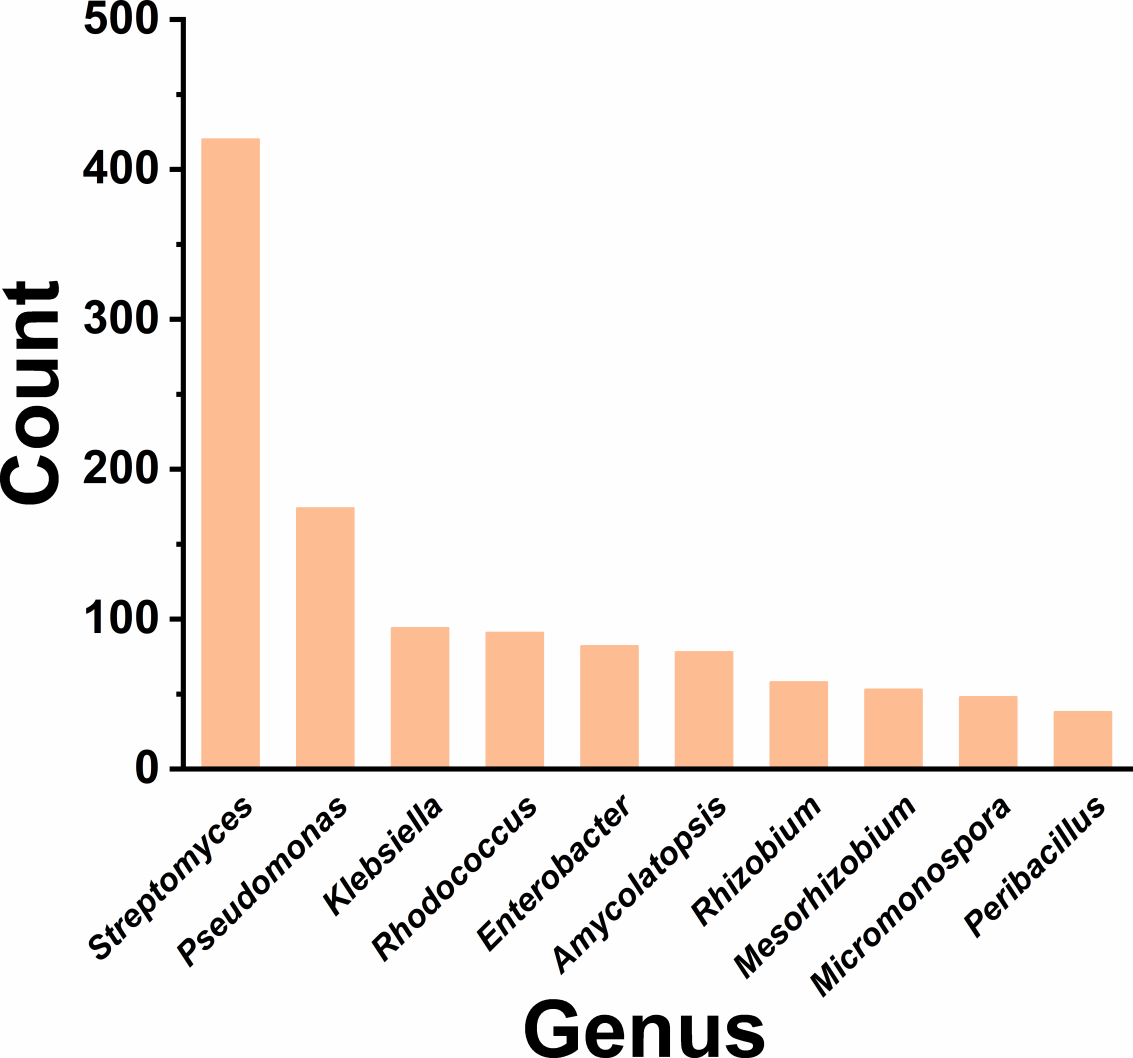
Distribution of CxyA homologs at the genus level (top 10 genera).

To explore the distribution of CxyA, an overall query using the CxyA protein sequence against all the available bacterial genomes with whole-genome assembly in the NCBI RefSeq database was performed. It was found that CxyA existed in 2,203 bacterial genomes. The major genera included *Streptomyces* (420), *Pseudomonas* (174), *Klebsiella* (94), *Rhodococcus* (91), *Enterobacter* (82), *Amycolatopsis* (78), *Rhizobium* (58), *Mesorhizobium* (53), *Micromonospora* (48), and *Peribacillus* (38) (Fig. 6). The conserved catalytic residues across these homologs strongly support their functional role in PCMX biotransformation, informing strategic microbial resource prospecting for bioremediation.

### Environmental implications

This study resolves critical uncertainties surrounding the environmental fate of PCMX—a pervasive antimicrobial contaminant—by elucidating its degradation mechanism in a newly isolated strain *Rhodococcus pyridinivorans* DMU114. The repeated isolation of *Rhodococcus* spp. in different labs, as well as in our experiments, underscores their crucial role as keystone degraders for PCMX detoxification, positioning them as prime candidates for bioaugmentation in wastewater treatment (22–26). We conclusively demonstrate that PCMX degradation initiates via *ortho*-hydroxylation (catalyzed by the novel flavin-dependent monooxygenase CxyAB) rather than dechlorination (23). This first-step hydroxylation generates 4-chloro-3,5-dimethylcatechol, followed by oxidative dechlorination to 2-hydroxy-3,5-dimethyl-[1,4]benzoquinone, providing new insights into our understanding of the environmental fate of PCMX (25). It also provides new insights into PCMX risks by taking into account the potential toxicity of its metabolites. The discovery of dual catechol cleavage pathways (C12O and C23O) in downstream processing highlights its metabolic diversity under natural conditions and suggests that the rate-limiting step for PCMX degradation is likely associated with upstream metabolism (42, 59). By mapping the enzyme CxyA and its phylogenetic prevalence across various strains, this work provides actionable insights for optimizing microbial consortia designed to target PCMX removal.

## Data Availability

The genome sequence of strain DMU114 has been deposited in the GenBank database with the accession number CP194257. Data will be made available on request.

## SUPPLEMENTAL MATERIAL

Supplemental material aem.00984-25-s0001.docx Tables S1 to S8; Fig. S1 to S17.

ASM does not own the copyrights to Supplemental Material that may be linked to, or accessed through, an article. The authors have granted ASM a non-exclusive, world-wide license to publish the Supplemental Material files. Please contact the corresponding author directly for reuse.
