# Supplementary material for: Elucidating the metabolic pathway and initial degradation gene for p-chloro-m-xylenol biodegradation in Rhodococcus pyridinivorans DMU114
Source: Appl Environ Microbiol. 2025 Aug 18;91(9):e00984-25. doi: 10.1128/aem.00984-25 (PMC12442393; doi:10.1128/aem.00984-25)
Supplement: Supplemental material — Tables S1 to S8; Fig. S1 to S17. [file aem.00984-25-s0001.docx]

**Supplementary materials**

**Elucidating the metabolic pathway and initial degradation gene for** ***p*-chloro-*m*-xylenol biodegradation in *Rhodococcus* *pyridinivorans* DMU114**

Liang Zhao^a^, Jia Shi^a^, Jingwei Wang^a^, Hao Zhou^b^, Dan Xu^a^, Qiao Ma^a,*^

^a^Institute of Environmental Systems Biology, College of Environmental Science and Engineering, Dalian Maritime University, Dalian 116026, China

^b^School of Chemical Engineering, Ocean and Life Science, Dalian University of Technology, Panjin 124221, China

**^*^Corresponding author: Qiao Ma**, e-mail: xiaoma0556@dlmu.edu.cn

| **Tables** | **8** |
| --- | --- |
| **Figures** | **17** |

Table S1 Primers for qRT-PCR assay

| Enzyme | Gene name | Primer name | Sequence |
| --- | --- | --- | --- |
| P450 | *rp1167* | 1167-QF | CGTACATATCGGTCCTGCCC |
|  |  | 1167-QR | GACGTGGACGGTCATTTCCT |
|  | *rp1183* | 1183-QF | CAACTCCGAACCGAACTGGA |
|  |  | 1183-QR | GCATCGAGTCGTGATAGCCA |
|  | *rp1290* | 1290-QF | CTCTCGTCGGTATTCCTGGC |
|  |  | 1290-QR | GAAGTTGGAGCCGAACCTGA |
|  | *rp1292* | 1292-QF | CATCGACCTCAGCAACCCTT |
|  |  | 1292-QR | GAGCTCGTGGTTCTTGTCCA |
|  | *rp3284* | 3284-QF | CACCATCTGCTGTTGCGTTT |
|  |  | 3284-QR | GAACGGCAACGACGTGAAG |
|  | *rp3400* | 3400-QF | TGTTGTCGACCCATTCCGAG |
|  |  | 3400-QR | GTACTCGTAGACCTGCGGTG |
|  | *rp3401* | 3401-QF | GAAGTTCGGTTACCTGCCGA |
|  |  | 3401-QR | TCACTTCGACTCACCTTCCG |
|  | *rp3498* | 3498-QF | AAGAGGCACCCATTGCCTAC |
|  |  | 3498-QR | GGTTCGACCATGTCCCTCAG |
|  | *rp3511* | 3511-QF | ACGGAGAAGAGCATCGCAAA |
|  |  | 3511-QR | CGCACCGAGGTCTTCTATCC |
|  | *rp3590* | 3590-QF | ACTGAAGACACTCACGCTCG |
|  |  | 3590-QR | TGAATGCCTTCTTCACCGCT |
|  | *rp3666* | 3666-QF | CGACTACATCTACCCGACGC |
|  |  | 3666-QR | AGAGTGGAACTCATGGCGTG |
|  | *rp4775* | 4775-QF | CTACATCGACCCGAACCAGG |
|  |  | 4775-QR | GCTGGACCACCATCTTCGAG |
|  | *rp4925* | 4925-QF | TTCGGGTGTCCTTCAACGAG |
|  |  | 4925-QR | AACGGGTGGATTCCGATAGC |
| C23O | *rp3503* | 3503-QF | CAACGTCGACGGTAAGTGGA |
|  |  | 3503-QR | ACGTACTTCTCGACCTCCCA |
| C12O | *rp3202* | 3202-QF | CTCGCTCGTCAAGGAGTTTG |
|  |  | 3202-QR | CGATGTCGAACCGGACCTT |
|  | *rp3659* | 3659-QF | GCGACCGACAAGTTCAAGTC |
|  |  | 3659-QR | TCACCTGGTGCTTCTGGATG |
|  | *rp3787* | 3787-QF | AGCTCATTCTGGATCCGCAG |
|  |  | 3787-QR | GGGTCGAGCACGAAGTTGTA |
|  | *rp3663* | 3663-QF | GAATACGGCCTCACCTACCG |
|  |  | 3663-QR | ACGTCCGGGTTCTTGAAGTC |
| Reference gene | *gyrB* | gyrB-QF | GATCTACATCGTGGAGGGCG |
|  |  | gyrB-QR | GACGTTGATGATCTTGCCGC |

Table S2 Primers for gene heterologous expression assay

| Gene | Primer name | Sequence |
| --- | --- | --- |
| pET-28a(+) plasmid | pET-28a-F | CTCGAGCACCACCACCACCA |
|  | pET-28a-R | GGATCCGCGACCCATTTGCT |
| *rp3510/3511* | 3510/1-CE-F | CAGCAAATGGGTCGCGGATCCATGACCCGTGCTGCCACCTGGA |
|  | 3510/1-CE-R | GTGGTGGTGGTGGTGCTCGAGTCATGCTGGCATTCGGGCCG |
| *rp3662/3663* | 3662/3-CE-F | CAGCAAATGGGTCGCGGATCCATGGATCAGCGCACACTCCGCAA |
|  | 3662/3-CE-R | GTGGTGGTGGTGGTGCTCGAGTTACTTGTAGAACGCGCTGACGT |
| *rp3666/3667* | 3666/7-CE-F | CAGCAAATGGGTCGCGGATCCATGACCAGCACCCTTTCGTGGCT |
|  | 3666/7-CE-R | GTGGTGGTGGTGGTGCTCGAGTCAGCCGGCCGGGGTGAACCGTT |

Table S3 Comparation analysis of the 16S rRNA genes with related strains

| Strain | Close strains | Similarity |
| --- | --- | --- |
| #1 | *Microbacterium arabinogalactanolyticum* | 99.32% |
| #2 | *Acinetobacter pittii* | 99.93% |
| #3 | *Stenotrophomonas maltophilia* | 99.18% |
| #4 | *Pseudomonas nitroreducens* | 100% |
| #5 | *Achromobacter denitrificans* | 99.63% |
| #6 | *Bacillus wiedmannii* | 100% |
| #7 | *Rhodococcus pyridinivorans* | 99.34% |

Table S4 PCMX degradation kinetics by first-order kinetic model analyses

| Concentration  (mg/L) | pH | Temperature  (°C) | Kinetic equation | *k* (h^−1^) | t_1/2_ (h) | r^2^ |
| --- | --- | --- | --- | --- | --- | --- |
| 20 | 5 | 30 | Unfitted | -- | -- | -- |
| 20 | 6 | 30 | C = 21.7729e^-0.0384t^ | 0.0384 | 18.0648 | 0.8307 |
| 20 | 7 | 30 | C = 21.0319e^-0.0488t^ | 0.0488 | 14.1922 | 0.9505 |
| 20 | 8 | 30 | C = 23.3618e^-0.0515t^ | 0.0515 | 13.4696 | 0.9482 |
| 20 | 9 | 30 | C = 23.8615e^-0.0334t^ | 0.0334 | 20.7281 | 0.7644 |
| 20 | 7 | 25 | C = 25.7608e^-0.0398t^ | 0.0398 | 17.4377 | 0.7925 |
| 20 | 7 | 35 | C = 22.2854e^-0.0709t^ | 0.0709 | 9.7764 | 0.9076 |
| 20 | 7 | 40 | C = 23.2107e^-0.0143t^ | 0.0143 | 48.4718 | 0.9638 |

Table S5 Summary of the oxygenase genes in strain DMU114 by RAST analysis

| **No.** | **Gene ID** | **Annotation** |
| --- | --- | --- |
| 1 | *rp74* | Putative monooxygenase |
| 2 | *rp136* | Oxidoreductase, 2OG-Fe(II) oxygenase family protein |
| 3 | *rp166* | Lignostilbene-alpha,beta-dioxygenase and related enzymes |
| 4 | *rp237* | Cyclohexanone monooxygenase |
| 5 | *rp307* | Dioxygenase, TauD/TfdA family |
| 6 | *rp311* | Monooxygenase, flavin-binding family |
| 7 | *rp325* | Nitrilotriacetate monooxygenase component A |
| 8 | *rp562* | Flavin-containing monooxygenase |
| 9 | *rp687* | Protocatechuate 3,4-dioxygenase alpha chain |
| 10 | *rp688* | Protocatechuate 3,4-dioxygenase beta chain |
| 11 | *rp1112* | Heme oxygenase |
| 12 | *rp1128* | Glyoxalase/bleomycin resistance protein/dioxygenase |
| 13 | *rp1161* | Oxidoreductase MSMEG_4685, 2OG-Fe(II) oxygenase family |
| 14 | *rp1162* | Oxidoreductase, 2OG-Fe(II) oxygenase family protein |
| 15 | *rp1191* | Putative oxidoreductase, nitronate monooxygenase family |
| 16 | *rp1267* | Flavin-dependent monooxygenase ArsO associated with arsenic resistance |
| 17 | *rp1313* | Putative dioxygenase |
| 18 | *rp1480* | Nitrilotriacetate monooxygenase component B |
| 19 | *rp1485* | Gentisate 1,2-dioxygenase |
| 20 | *rp1496* | Gentisate 1,2-dioxygenase |
| 21 | *rp2138* | Putative oxidoreductase, nitronate monooxygenase family |
| 22 | *rp2493* | Alkane-1 monooxygenase |
| 23 | *rp2820* | Nitrilotriacetate monooxygenase component B |
| 24 | *rp2905* | Possible flavin binding monooxygenase |
| 25 | *rp2941* | Flavohemoglobin / Nitric oxide dioxygenase |
| 26 | *rp2976* | 4-Hydroxyphenylpyruvate dioxygenase |
| 27 | *rp3039* | Glyoxalase/bleomycin resistance protein/dioxygenase |
| 28 | *rp3129* | Nitrilotriacetate monooxygenase component B |
| 29 | *rp3130* | Luciferase-like monooxygenase YhbW |
| 30 | *rp3153* | Alkane-1 monooxygenase |
| 31 | ***rp3202*** | **Catechol 1,2-dioxygenase** |
| 32 | *rp3205* | 2,3-Dihydroxybiphenyl 1,2-dioxygenase |
| 33 | *rp3210* | 3-Ketosteroid-9-alpha-monooxygenase, oxygenase component |
| 34 | *rp3271* | Glyoxalase/bleomycin resistance protein/dioxygenase |
| 35 | *rp3400* | Cytochrome P450 monooxygenase |
| 36 | ***rp3503*** | **Catechol 2,3-dioxygenase** |
| 37 | *rp3546* | Cyclohexanone monooxygenase |
| 38 | *rp3576* | Homogentisate 1,2-dioxygenase |
| 39 | *rp3597* | 4-Hydroxyphenylpyruvate dioxygenase |
| 40 | ***rp3659*** | **Catechol 1,2-dioxygenase** |
| 41 | *rp3663* | 4-Hydroxyphenylacetate 3-monooxygenase |
| 42 | *rp3668* | Benzoate 1,2-dioxygenase alpha subunit |
| 43 | *rp3669* | Benzoate 1,2-dioxygenase beta subunit |
| 44 | *rp3767* | 4-Hydroxyphenylacetate 3-monooxygenase |
| 45 | ***rp3787*** | **Catechol 1,2-dioxygenase** |
| 46 | *rp3887* | Cysteine dioxygenase |
| 47 | *rp3968* | FMNH2-dependent alkanesulfonate monooxygenase |
| 48 | *rp3974* | Monooxygenase, flavin-binding family |
| 49 | *rp4104* | Nitrilotriacetate monooxygenase component A |
| 50 | *rp4190* | Putative monooxygenase |
| 51 | *rp4212* | NADH:quinone reductase, NO nitronate monooxygenase activity |
| 52 | *rp4216* | Antibiotic biosynthesis monooxygenase domain protein |
| 53 | *rp4285* | Flavin-containing monooxygenase |
| 54 | *rp4337* | Aliphatic sulfonate monooxygenase family, FMNH2- or F420-dependent |
| 55 | *rp4380* | NADH:quinone reductase, NO nitronate monooxygenase activity |
| 56 | *rp4401* | Cyclohexanone monooxygenase |
| 57 | *rp4536* | 3-Ketosteroid-9-alpha-monooxygenase, oxygenase component |
| 58 | *rp4537* | Flavin-dependent monooxygenase, oxygenase subunit HsaA |
| 59 | *rp4539* | Iron-dependent extradiol dioxygenase |
| 60 | *rp4540* | Flavin-dependent monooxygenase, reductase subunit HsaB |
| 61 | *rp4570* | Monooxygenase IpdC |
| 62 | *rp4580* | 3-Ketosteroid-9-alpha-monooxygenase, oxygenase component |
| 63 | *rp4589* | 2,3-Dihydroxybiphenyl 1,2-dioxygenase |
| 64 | *rp4596* | Nitrilotriacetate monooxygenase component B |
| 65 | *rp4610* | Steroid C27-monooxygenase |
| 66 | *rp4622* | Putative oxidoreductase, nitronate monooxygenase family |
| 67 | *rp4624* | Putative oxidoreductase, nitronate monooxygenase family |
| 68 | *rp4645* | Siderophore biosynthesis protein, monooxygenase |
| 69 | *rp4678* | Alpha-ketoglutarate-dependent dioxygenase AlkB |
| 70 | *rp4760* | Flavin-dependent monooxygenase ArsO associated with arsenic resistance |
| 71 | *rp4771* | Steroid C27-monooxygenase |
| 72 | *rp4773* | Steroid-monooxygenase |
| 73 | *rp4867* | Nitrilotriacetate monooxygenase component A |
| 74 | *rp4964* | Dioxygenase, TauD/TfdA family |
| 75 | *rp5004* | Flavohemoglobin / Nitric oxide dioxygenase |

Table S6 Summary of the P450 enzyme genes in strain DMU114

| **No.** | **Gene ID** | **Annotation** |
| --- | --- | --- |
| 1 | *rp1167* | Cytochrome P450 |
| 2 | *rp1183* | Putative cytochrome P450 |
| 3 | *rp1290* | Cytochrome P450 |
| 4 | *rp1292* | Putative cytochrome P450 hydroxylase |
| 5 | *rp3284* | Cytochrome P450 CYP136 |
| 6 | *rp3400* | Cytochrome P450 monooxygenase |
| 7 | *rp3401* | Putative cytochrome P450 hydroxylase |
| 8 | *rp3498* | Cytochrome P450 |
| 9 | *rp3511* | Cytochrome P450 |
| 10 | *rp3590* | Cytochrome P450 |
| 11 | *rp3666* | Cytochrome P450 |
| 12 | *rp4775* | Lanosterol 14-alpha demethylase @ Cytochrome P450 51 |
| 13 | *rp4925* | Putative cytochrome P450 hydroxylase |

Table S7 Gene information of the identified two clusters

| Cluster | ID | Gene | Annotation |
| --- | --- | --- | --- |
| **Cluster**  **I** | *rp3657* | *catC* | Muconolactone isomerase |
|  | *rp3658* | *catB* | Muconate cycloisomerase |
|  | *rp3659* | *catA* | Catechol 1,2-dioxygenase |
|  | *rp3660* | *pcaR* | Pca regulon regulatory protein PcaR |
|  | *rp3661* | *araC* | Transcriptional regulator, AraC family |
|  | *rp3662* | *pheA2* | NADH-FMN oxidoreductase |
|  | *rp3663* | *hpaB* | 4-Hydroxyphenylacetate 3-monooxygenase |
|  | *rp3664* | *-* | Hypothetical protein |
|  | *rp3665* | *araC* | Hypothetical protein |
|  | *rp3666* | P450 | Cytochrome P450 |
|  | *rp3667* | *dmpP* | 2-Polyprenylphenol hydroxylase and related flavodoxin oxidoreductases / CDP-6-deoxy-delta-3,4-glucoseen reductase-like |
|  | *rp3668* | *benA* | Benzoate 1,2-dioxygenase alpha subunit |
|  | *rp3669* | *benB* | Benzoate 1,2-dioxygenase beta subunit |
|  | *rp3670* | *benC* | Oxidoreductase FAD-binding domain protein |
|  | *rp3671* | *benD* | 1,2-Dihydroxycyclohexa-3,5-diene-1-carboxylate dehydrogenase |
| **Cluster**  **II** | *Rp3503* | *--* | Catechol 2,3-dioxygenase |
|  | *rp3504* | *mhpF* | Acetaldehyde dehydrogenase, acetylating |
|  | *rp3505* | *dmpG* | 4-Hydroxy-2-oxovalerate aldolase |
|  | *rp3506* | *hpaE* | 5-Carboxymethyl-2-hydroxymuconate semialdehyde dehydrogenase |
|  | *rp3507* | *--* | Putative short-chain dehydrogenase |
|  | *Rp3508* | *xylH* | 2-Hydroxymuconate tautomerase-like protein / 2-hydroxymuconate Tautomerase-like protein |
|  | *rp3509* | *nphA* | NADH-FMN oxidoreductase |
|  | *rp3510* | *mphP* | Probable phenol hydrolase |
|  | *rp3511* | P450 | Cytochrome P450 |
|  | *rp3512* | *araC* | Transcriptional regulator, AraC family |
|  | *rp3513* | *mmgC* | Acyl-CoA dehydrogenase, short-chain specific |
|  | *rp3514* | *xylH* | 4-Oxalocrotonate tautomerase |
|  | *rp3515* | *dmpH* | 4-Oxalocrotonate decarboxylase |
|  | *rp3516* | *hpaH* | 2-Oxo-hepta-3-ene-1,7-dioic acid hydratase |
|  | *rp3517* | *iclR* | Transcriptional regulator, IclR family |

Table S8 Comparison of CxyA and its related proteins

| Accession number | Protein | Gene | Organism | Identity to CxyA（%） |
| --- | --- | --- | --- | --- |
| Q8GMG6 | (3S)-3-Amino-3-(3-chloro-4-hydroxyphenyl)propanoyl-[peptidyl-carrier protein SgcC2] monooxygenase | *sgcC* | *Streptomyces globisporus* | 64.68 |
| Q6F4M8 | 4-Nitrophenol 4-monooxygenase | *npcA* | *Rhodococcus opacus* | 55.62 |
| A0A140NG21 | 4-Hydroxyphenylacetate 3-monooxygenase | *hpaB* | *Escherichia coli* BL21(DE3) | 55.62 |
| Q57160 | 4-Hydroxyphenylacetate 3-monooxygenase | *hpaB* | *Escherichia coli* | 55.23 |
| A0A520FDW1 | Pyrrole-2-carboxylate monooxygenase | *--* | *Rhodococcus* sp. | 33.94 |
| Q5SJP8 | 4-Hydroxyphenylacetate 3-monooxygenase | *--* | *Thermus thermophilus* ATCC 27634 | 33.55 |
| I0WZP1 | 4-Hydroxyphenylacetate 3-hydroxylase | *--* | *Rhodococcus opacus* RKJ300 | 25.49 |
| A9A1Y2 | Vinylacetyl-CoA delta-isomerase | *--* | *Nitrosopumilus maritimus* SCM1 | 23.92 |
| Q471I2 | 2,4,6-Trichlorophenol monooxygenase | *tcpA* | *Cupriavidus necator* JMP 134 | 23.83 |
| Q53008 | Chlorophenol monooxygenase | *hadA* | *Ralstonia pickettii* | 22.41 |

Figure S1 The enrichment process of PCMX-degrading consortium.

Figure S2 Dynamics of the consortium bacterial structure in the enrichment process at the phylum level. B-LB meant that the consortium B was cultured in LB medium for 24 h.

Figure S3 PCMX degradation performance by strain #7 and its combination with other strains.

Figure S4 *Rhodococcus* sp. DMU114 and its degradation characteristics. (a) Morphology of strain DMU114 on the solid plate and the cell morphology observed by scanning electron microscope. (b) Phylogenetic tree of strain DMU114 and related *Rhodococcus* strains.

Figure S5 Degradation performance of strain DMU114 towards different concentrations of PCMX.

Figure S6 Effects of different parameters on PCMX degradation in strain DMU114. (a) Temperature. (b) pH value.

Figure S7 The degradation potential of strain DMU114 towards PCMX derivatives. UPLC analysis was performed on Day 1 and Day 6.

Figure S8 Identification of the metabolic intermediates of PCMX degradation in strain DMU114.

Figure S9 Identification of the metabolic intermediate of PCMX degradation in strain DMU114 with the addition of ring-cleavage inhibitor 2,2'-bipyridyl.

Figure S10 Proposed PCMX biodegradation pathway in strain DMU114.


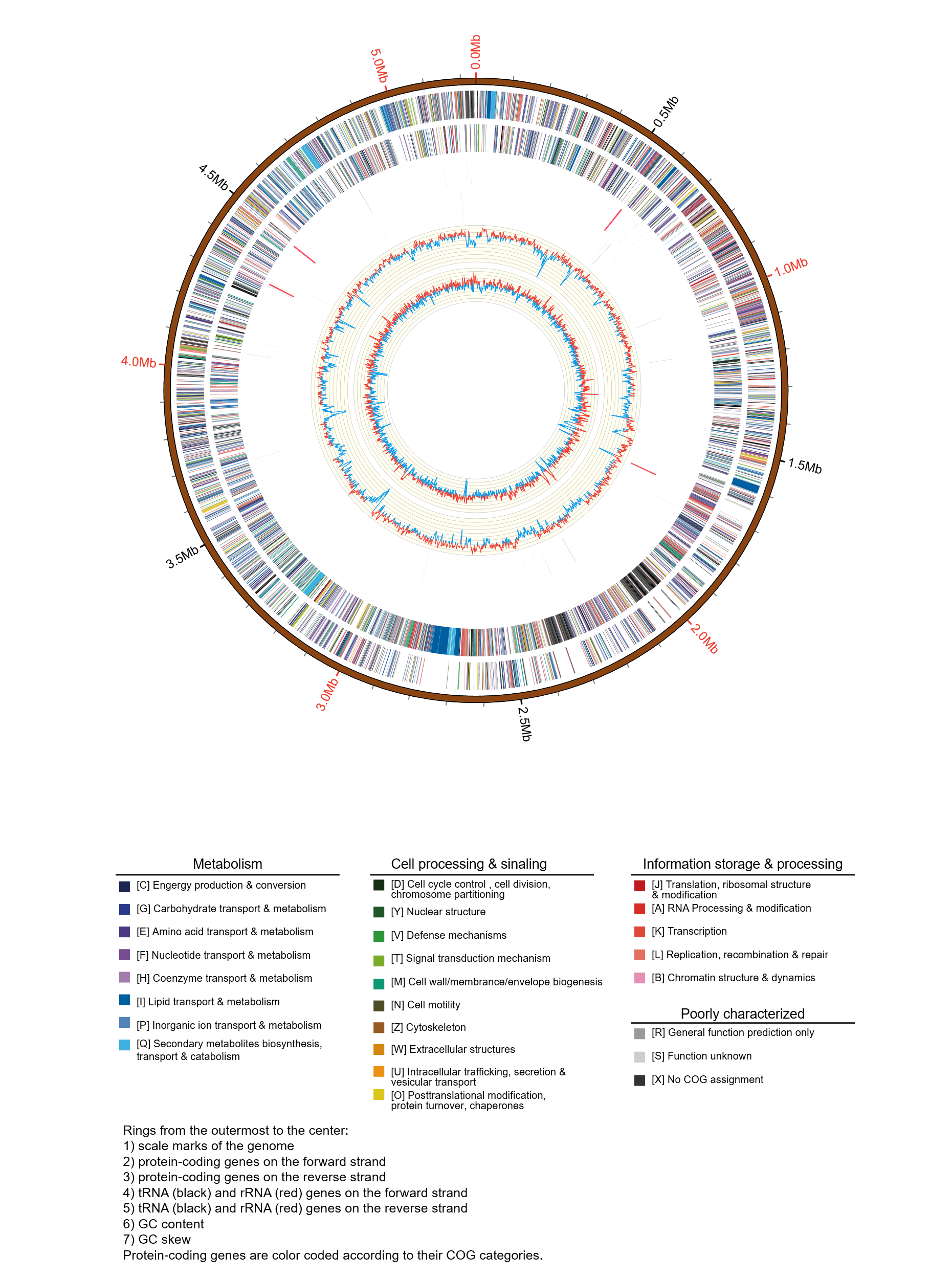


Figure S11 Genomic circle map of the chromosome DNA of strain DMU114.

Figure S12 Genome analysis of strain DMU114. (a) Gene numbers are annotated by NR, Swiss-Prot, COG, GO, and KEGG databases. (b) KEGG analysis at level 3.

Figure S13 PCMX biodegradation by induced and uninduced resting cells of strain DMU114.

Figure S14 Bacterial growth and PCMX degradation in the presence of P450 enzyme inhibitor 1-aminobenzotriazole (a) and piperonylbutoxide (b).

Figure S15 NMR analyses of the CxyAB-catalyzed product. (a) 1H NMR; (b) HSQC.


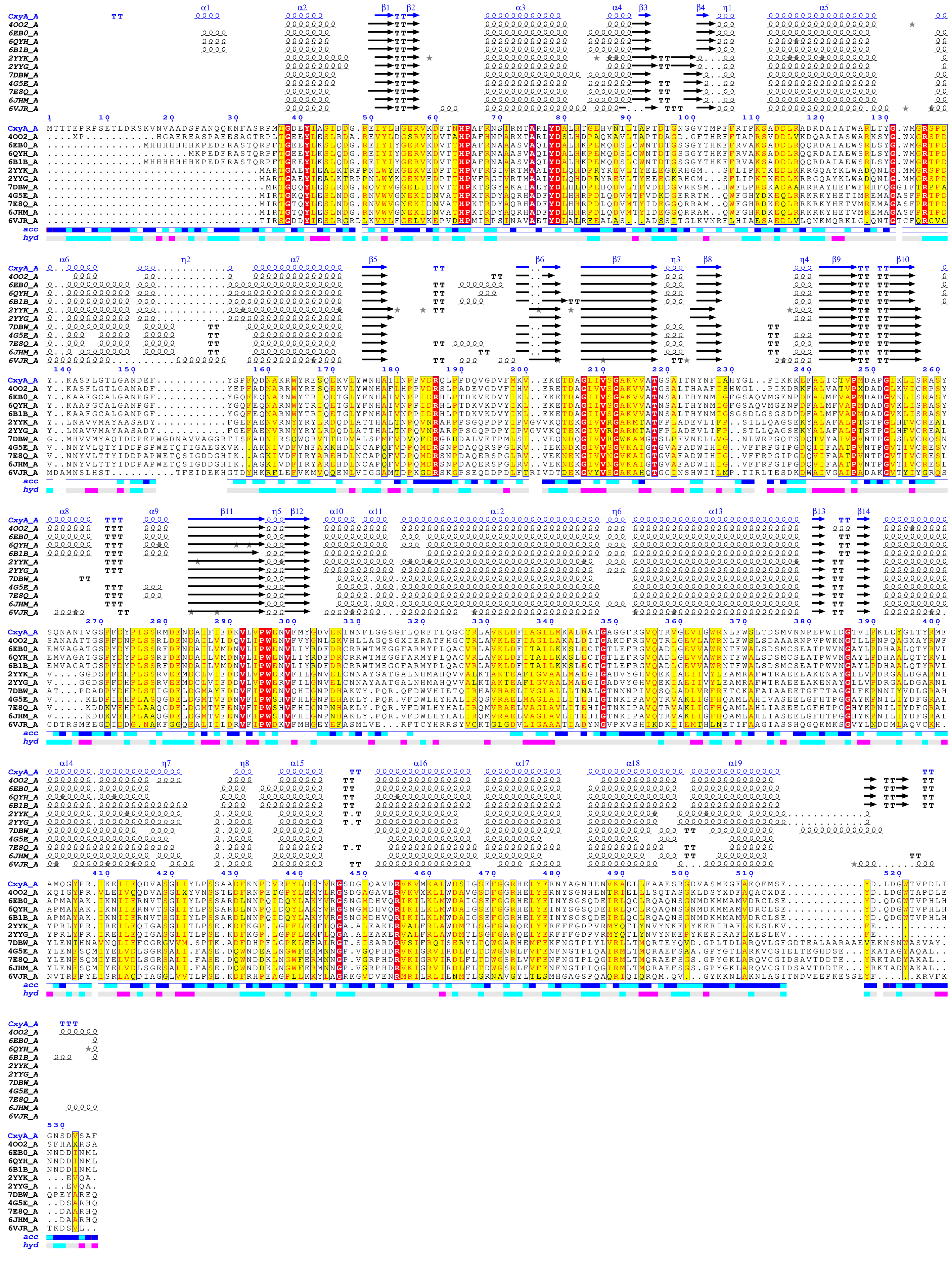


Figure S16 Alignment of CxyA and its related proteins from the PDB database. The program identifies α-helices (shown by medium squiggles), π-helices (large squiggles), β-strands (arrows), strict α-turns (letters), and β-turns (letters) from the 3D structure. The relative accessibility, calculated by DSSP in the previous step, is shown by a blue-colored bar below the sequence. White is buried (A < 0.1), cyan is intermediate (0.1 ≤ A ≤ 0.4), blue is accessible (0.4<A≤1), and blue with red edges is highly exposed (A > 1). A red box means that relative accessibility is not calculated for the residue, because it is truncated. Hydropathy is calculated from the sequence according to the Kyte & Doolittle algorithm. It is shown by a second bar below the accessibility: pink is hydrophobic(H > 1.5), grey is intermediate (-1.5 ≤ H ≤ 1.5), and cyan is hydrophilic (H < -1.5).


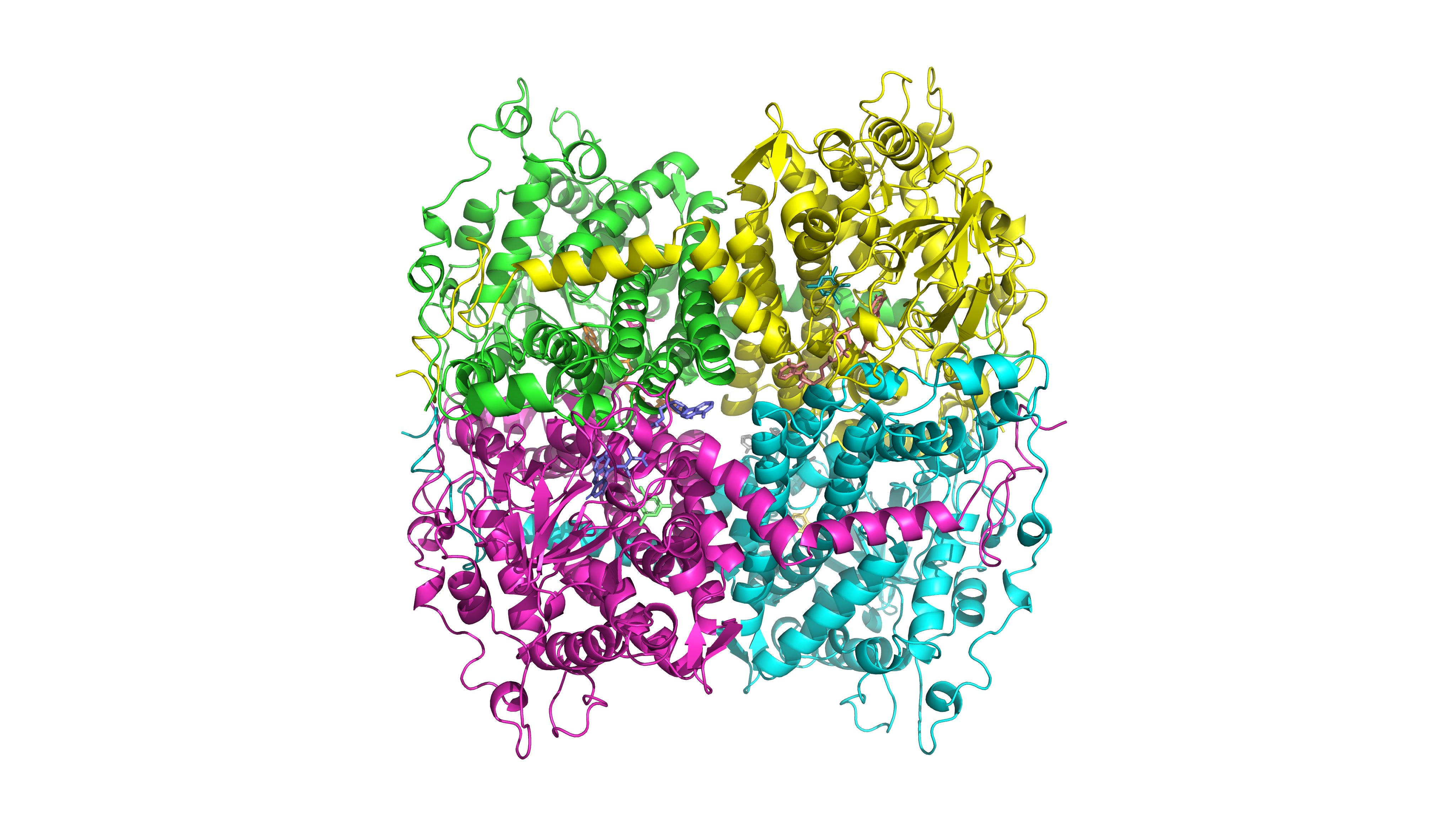

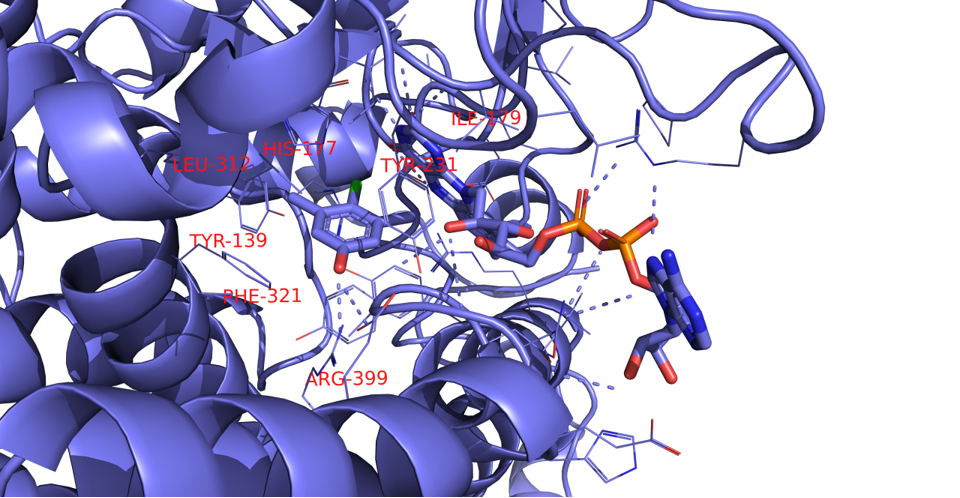


Figure S17 Structural prediction of CxyA (left) and CxyA-PCMX complex (right)
